# Refractory Acute Myocardial Infarction Cardiogenic Shock Due to Left Ventricular Outflow Tract Obstruction

**DOI:** 10.1016/j.jaccas.2025.104452

**Published:** 2025-08-13

**Authors:** Oludamilola Akinmolayemi, Frans J. Beerkens, Vivian Hu, Apurva Sharma, Jason Feinman, Syed Zain Ali, Sunny Goel, Gregory W. Serrao, Martin E. Goldman, Anuradha Lala

**Affiliations:** a Mount Sinai Fuster Heart Hospital, New York, New York, USA; b Department of Medicine, Icahn School of Medicine at Mount Sinai, New York, New York, USA; c Hackensack University Medical Center, Hackensack, New Jersey, USA; d New York University School of Medicine, New York, New York, USA; e Mount Sinai Heart, Mount Sinai South Nassau, Oceanside, New York, USA

**Keywords:** cardiogenic shock, echocardiography, left ventricular outflow tract obstruction, mechanical circulatory support, myocardial infarction

## Abstract

**Background:**

Optimal management of cardiogenic shock (CS) after acute myocardial infarction (AMI) frequently consists of inotropes and consideration of a mechanical circulatory support. However, this initial approach may be counterproductive in some CS phenotypes.

**Case Summary:**

A 75-year-old female presented with AMI of the left anterior descending artery and underwent successful revascularization. Initial postprocedural management including intra-aortic balloon pump and inotropes paradoxically worsened CS. Prompt echocardiography illustrated an unrecognized left ventricular outflow tract obstruction (LVOTO) due to an apical aneurysmal infarct and compensatory hypercontractile basal segments. Echocardiography-guided weaning of the intra-aortic balloon pump and inotropes resulted in improved hemodynamics.

**Discussion:**

Dynamic LVOTO is an underrecognized mechanical complication of anterior-wall AMI-CS. Clinical awareness and echocardiography can help identify anatomical predisposition to LVOTO and direct appropriate hemodynamic support.

**Take-Home Message:**

This case highlights the importance of recognizing LVOTO in AMI-CS where afterload-reducing mechanical circulatory support and inotropes can counterintuitively worsen hemodynamics.

## Graphical Abstract

**Figure:**
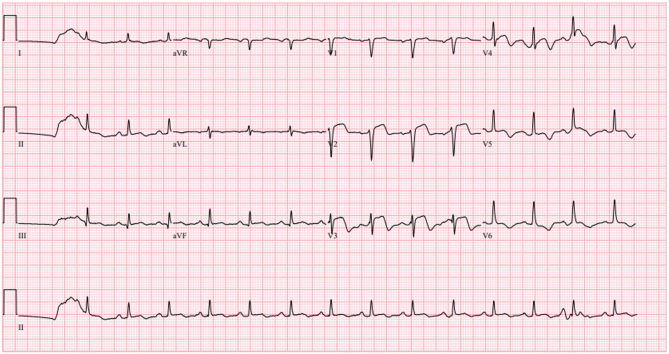

## History of presentation

**Figure 1:**
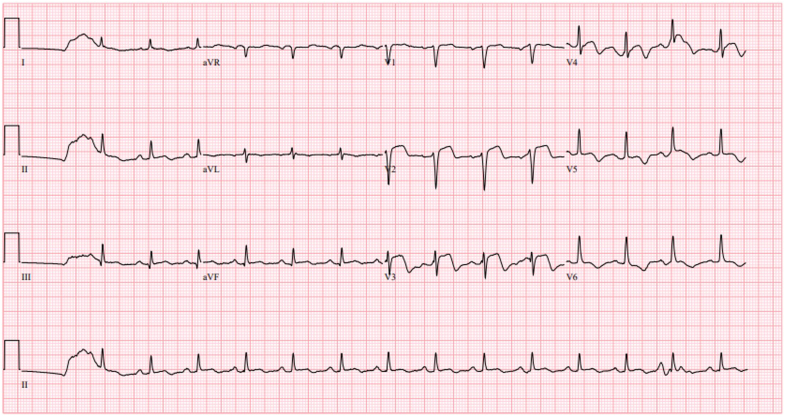
Electrocardiogram on Presentation Electrocardiogram showing normal sinus rhythm and anteroseptal ST-segment elevation with reciprocal T-wave inversion in lateral leads.

A 75-year-old female presented to an outside hospital emergency department with initial symptoms of progressive worsening chest tightness, nausea, and vomiting for 2 days with associated dyspnea on minimal exertion and orthopnea. Laboratory findings included a troponin-I trend of 10.69 to 20.4 ng/mL (reference 0-14 ng/L), creatine kinase-MB of 42.4 ng/mL (reference 0.6-6.3 ng/mL), and lactate of 5.1 mmol/L (reference 0.50-0.99 mmol/L). Electrocardiogram was notable for normal sinus rhythm and anteroseptal ST-segment elevation with reciprocal T-wave inversions in the lateral leads (Figure 1). Left-heart catheterization demonstrated 1-vessel disease, with 90% calcific stenosis in the proximal left anterior descending artery (LAD), 95% thrombotic stenosis of the mid LAD, and minor luminal irregularities in the left circumflex and right coronary arteries (Videos 1 and 2). She underwent percutaneous coronary intervention with drug-eluting stents to the proximal and mid LAD complicated by slow flow and sustained ventricular tachycardia (VT) requiring electrical cardioversion. With vasoactive support to maintain blood pressure, TIMI flow grade 3 was ultimately achieved (Video 3). An intra-aortic balloon pump (IABP) was placed under fluoroscopic guidance and maintained at 1:1 augmentation for acute myocardial cardiogenic shock (AMI-CS). Due to limited resources at this hospital, she was transferred to our quaternary care institution for further management.

## Past medical history

The patient has a relevant past medical history of hypertension, hyperlipidemia, and overweight (body mass index of 27.7 kg/m^2^).

## Differential diagnosis

The differential diagnosis of persistent AMI-CS despite inotropic support and mechanical circulatory support considered in this patient included acute left ventricular systolic dysfunction, acute right ventricular dysfunction, AMI-related mechanical complications, and dynamic left ventricular outflow tract obstruction (LVOTO).

## Investigations

**Figure 2:**
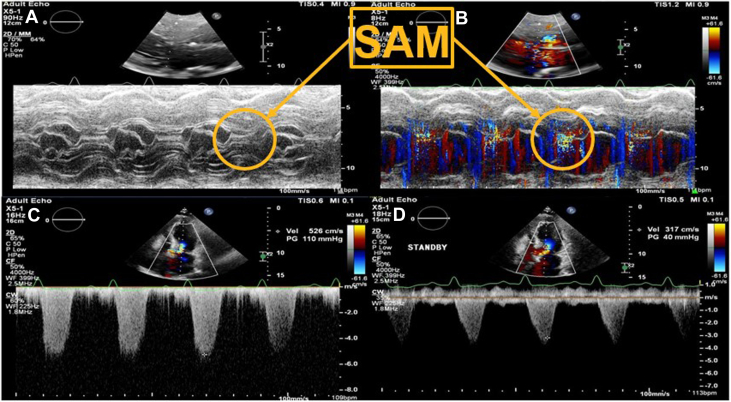
Transthoracic Echocardiogram with IABP at 1:1 Augmentation and High-Dose Vasopressors (A) M-mode showing SAM of mitral valve. (B) Color Doppler M-mode showing SAM of mitral valve. (C) Continuous-wave Doppler showing severe LVOTO. (D) Continuous-wave Doppler showing improvement of LVOTO while IABP on standby. IABP = intra-aortic balloon pump; LVOTO = left ventricular outflow tract obstruction; SAM = systolic anterior motion.

Upon arrival, she had a mean arterial pressure of 66 mm Hg while receiving norepinephrine at 0.24 μg/kg/min in addition to the IABP at 1:1 support. She had a normal mental status and appeared comfortable on 3-L nasal cannula. Physical examination was notable for elevated jugular venous pressure, a III/VI systolic murmur at the left sternal border, and warm and well-perfused extremities with no lower-extremity edema. Electrocardiogram showed normal sinus rhythm, left posterior fascicular block, anterior Q-waves, and T-wave inversions in the inferolateral leads. A limited point-of-care ultrasound showed severe left ventricular systolic dysfunction with diffuse severe hypokinesis in the apical and mid-segments, but with hypercontractility of the basal segments. In addition, there was suspicion of apical thrombus. Lactate improved from 5.1 to 2.4 mmol/L without any additional intervention; however, cardiac hemodynamics by pulmonary artery catheter demonstrated elevated filling pressures and low cardiac output (Table 1). Her course was complicated by worsening hypotension, requiring an increased dose of norepinephrine and the addition of epinephrine. Her lactate level increased to 6.4 mmol/L (Table 1). A transthoracic echocardiogram (TTE) was notable for a decreased left ventricular ejection fraction (LVEF) of 30%, localized mild basal septum hypertrophy of 13 mm, abnormalities in anterior-wall motion, apical aneurysm, systolic anterior motion (SAM) of the mitral valve, and a dynamic LVOTO with a left ventricular outflow (LVOT) peak gradient of 110 mm Hg and peak velocity of 5.3 m/s (Videos 4 and 5, Figures 2A and 2B, Table 1). However, with the IABP on standby, the LVOT peak gradient and velocity decreased to 40 mm Hg and 3.2 m/s, respectively (Figures 2C and 2D, Table 1). Ultrasound-enhancing agent-augmented images confirmed the presence of multiple apical thrombi (Video 6).

**Table 1:** Hemodynamics Trend During Management of Acute Myocardial Infarction Cardiogenic Shock

	HD 1	HD 2		HD 3				HD 5
Vasopressors (μg/kg/min) and normal saline	–	Norepinephrine 0.24	Norepinephrine 0.10	Norepinephrine 0.34 Epinephrine 0.3	Norepinephrine 0.25 Epinephrine 0.3 Vasopressin 1.8 U/kg/min	Norepinephrine 0.24 Vasopressin 2.4 U/kg/min	500 mL normal saline	–
IABP augmentation	–	1:1	1:1	1:1	1:1	1:2	1:2	–
Lactate (mmol/L)	5.1	2.4 → 1.2	1.2 → 2.4	2.4 → 6.4	6.4 → 4.8	4.8	1.0	0.6
Cardiac hemodynamics								
CVP (mean—mm Hg)	–	15	14	14	12	10	16	4
PAP (systolic/diastolic—mm Hg)	–	50/25	50/26	51/21	53/16	–	50/22	35/7
PCWP (mean—mm Hg)	–	26	26	21	16	–	22	7
CO (L/min)	–	2.0	2.7	3.8	4.1	–	3.6	4.4
CI (L/min/m^2^)	–	1.1	1.5	2.1	2.2	–	1.2	2.4
SVR (dynes × s/cm^5^)	–	2,990	1,575	1,066	1,052	–	1,323	1,460
SvO_2_ (%)	–	–	60	60	67	–	63	63
Echocardiographic parameters								
LVEF (%)	–	25	–	30	–	–	30-35	–
LVOT peak gradient (mm Hg)	–	31	–	110 40 (IABP on standby)	–	–	71 20 (IABP on standby)	–
LVOT peak velocity (m/s)	–	2.8	–	5.3 3.2 (IABP on standby)	–	–	4.2 2.3 (IABP on standby)	–

CI = cardiac index; CO = cardiac output; CVP = central venous pressure; HD = hospital day; IABP = intra-aortic balloon pump; LVEF = left ventricular ejection fraction; LVOT = left ventricular outflow tract; PAP = pulmonary artery pressure; PCWP = pulmonary capillary wedge pressure; SvO_2_ = mixed venous oxygen saturation; SVR = systemic vascular resistance.

## Management

**Figure 3:**
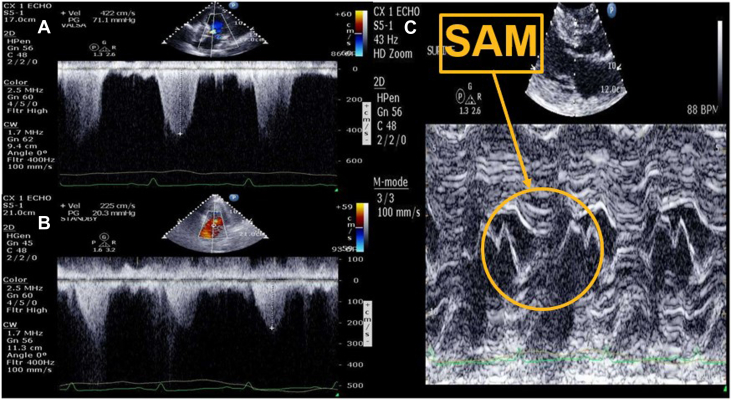
Transthoracic Echocardiogram With IABP at 1:2 Augmentation, Vasopressors Weaned Off, and IVF Administration (A) Continuous-wave Doppler showing LVOTO. (B) Continuous-wave Doppler showing improvement of LVOTO while IABP on standby. (C) M-mode showing SAM. IABP = intra-aortic balloon pump; IVF = intravenous fluids; LVOTO = left ventricular outflow tract obstruction; SAM = systolic anterior motion.

Given concern for dynamic LVOTO, epinephrine was cross-titrated to vasopressin, norepinephrine was weaned down, and IABP augmentation decreased to 1:2. Her lactate level correspondingly improved to 4.8 mmol/L (Table 1). Over the course of 36 hours, all vasopressors were weaned off, intravenous fluids were administered, and the IABP maintained at 1:2 augmentation, after which her lactic acidosis resolved. Repeat TTE demonstrated an LVOT peak gradient of 71 mm Hg with IABP at 1:2 augmentation and 20 mm Hg, although still with SAM, with IABP on standby (Table 1, Figure 3). The IABP was ultimately removed, and she remained hemodynamically stable. She was discharged on hospital day 9 with heart failure guideline-directed medical therapy (sacubitril-valsartan 24-26 mg twice daily, empagliflozin 10 mg daily, metoprolol succinate 25 mg daily, and spironolactone 25 daily), warfarin, clopidogrel, and a wearable cardioverter defibrillator.

## Outcome and follow-up

**Figure 4:**
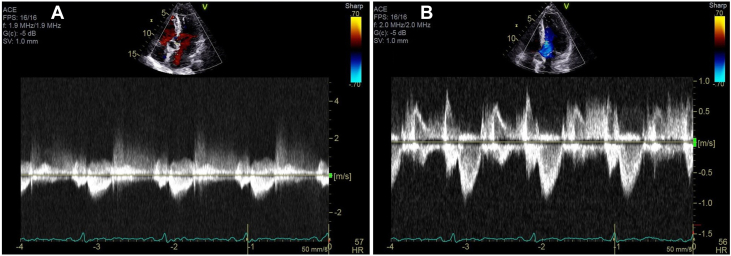
Transthoracic Echocardiogram at 1-Month Follow-Up (A) Continuous-wave Doppler showing LVOTO and (B) pulsed-wave Doppler showing resolution of LVOTO. LVOTO = left ventricular outflow tract obstruction.

She followed up with her local cardiologist 1 month after discharge where she reported feeling well overall and with good functional status. Repeat TTE showed improved LVEF to 55% to 60%, no LVOTO, and resolution of left ventricular thrombi (Videos 7 and 8, Figures 4A and 4B).

## Discussion

We present a case of an LAD-related AMI-CS complicated by dynamic LVOTO, which in turn worsened with IABP and escalating inotropic pharmacotherapy. It further demonstrates the importance of clinical awareness and timely reassessment of the effect of critical care interventions in refractory AMI-CS with readily available tools such as echocardiography.

Although traditionally defined in patients with hypertrophic cardiomyopathy, dynamic LVOTO is also a common yet highly underrecognized cause of cardiogenic shock in other clinic scenarios including AMI, takotsubo cardiomyopathy, left ventricular hypertrophy, mitral valve abnormalities, and after mitral or aortic valve operative repair.^1^, ^2^, ^3^

The pathophysiology of the dynamic LVOTO in this patient involves infarction of the portion of the left ventricle supplied by the LAD, with preservation of other regions. Specifically, obstruction of the proximal LAD leads to anteroseptal and apical akinesis, causing distortion of normal ventricular geometry and resulting in compensatory basal hyperkinesis as well as narrowing of the LVOT during mid-systole.^2^ Acceleration of blood flow through the LVOT creates a venturi effect on the anterior mitral leaflet, leading to SAM of the mitral valve, which further exacerbates LVOTO.^2^ In addition, dynamic LVOTO may result from other geometric and structural distortions of the left ventricle, including left ventricular hypertrophy, mitral apparatus abnormalities, acute aortoseptal angle, apical ballooning, as well as hemodynamic changes such as vasoplegia, reduced intravascular volume, tachycardia, and hypercontractility.^4^ Inotropic agents like dobutamine can notably also induce transient SAM and LVOTO, as seen in approximately 17% of healthy individuals during stress echocardiography without any clinical significance.^5^

LVOTO is often an unrecognized mechanical complication of AMI which requires prompt recognition and should be considered in patients with worsening hemodynamics despite conventional management. Understanding the anatomical distribution of myocardial infarction can help raise suspicion for LVOTO, particularly in the case of LAD as the culprit artery. However, recognition is best by echocardiography as it overcomes limitations of physical examination by exactly defining the ventricular geometry, LVOTO morphology and velocity, and further evaluating the mitral valve for duration and severity of SAM.^4^ It can also evaluate for other mechanical complications of AMI such as papillary muscle rupture, ventricular septal or free-wall rupture, and ventricular aneurysm. A pulmonary artery catheter, on the contrary, can be misleading during management, as a decreased cardiac output may be due to obstruction instead of ventricular dysfunction, and an elevated wedge pressure may reflect mitral regurgitation and SAM instead of congestion.

**Figure 5:**
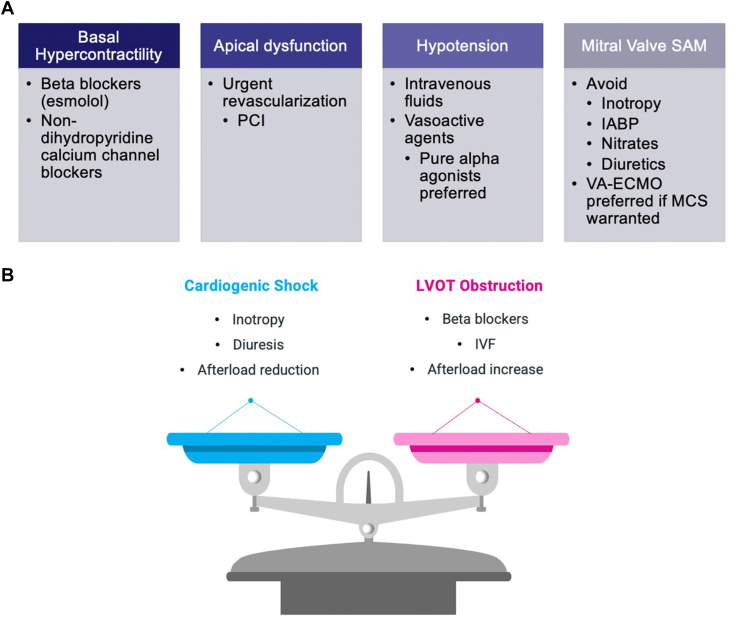
Management of Cardiogenic Shock (A) Based on structural and hemodynamic LVOTO causes. (B) Opposing treatments of LVOTO compared with usual management of cardiogenic shock. IABP = intra-aortic balloon pump; IVF = intravenous fluids; LVOTO = left ventricular outflow tract obstruction; MCS = mechanical circulatory support; PCI = percutaneous coronary intervention; VA-ECMO = veno-arterial extracorporeal membrane oxygenation.

Recognizing LVOTO as a cause of refractory CS is crucial, as standard therapies for AMI-CS, including inotropes and diuretics, can worsen LVOTO, ischemia, and even cause wall rupture (Figure 5A).^6^ Instead, treatment should be targeted to the geometric and hemodynamic causes of LVOTO (Figure 5B). Afterload reduction with temporary mechanical circulatory support devices such as an IABP must also be used with caution in AMI-CS with LVOTO. Although the reduced afterload improves diastolic coronary flow, which can address coronary artery intervention complications including slow and no reflow or arrhythmias, it can paradoxically worsen CS by exacerbating SAM of the mitral valve and LVOTO. If LVOTO is ultimately recognized as the cause of worsening or refractory cardiogenic shock, a prompt and careful transition to vasoconstrictive agents and withdrawal of inotropic and afterload-reducing agents including IABP can lead to amelioration and even resolution of shock as illustrated in this case.

Current American society guideline provides a Class I recommendation for implantable cardioverter-defibrillator as a secondary prevention intervention in case of sudden cardiac death in patients with ischemic heart disease and hemodynamically unstable VT without a reversible cause.^7^ Our patient experienced ischemia-driven sustained VT intraprocedure, requiring electrical cardioversion; however, the culprit artery was successfully revascularized, making this recommendation for implantable cardioverter-defibrillator not applicable. After extensive deliberation between the critical care and electrophysiology teams, a wearable cardioverter defibrillator was ultimately recommended as a temporary secondary prevention intervention of sudden cardiac death, particularly in the presence of decreased LVEF, with a plan to re-evaluate in the outpatient setting.

## Conclusions

Our case illustrates that in patients with initial presentation of AMI-CS, immediate management includes providing circulatory support with pharmacotherapy and mechanical circulatory devices as appropriate. However, in a situation of worsening or refractory CS despite appropriate guideline-directed management, a thorough clinical assessment including timely echocardiography with the awareness that LVOTO can be present in LAD-related AMI can be instrumental in re-directing conventional management.

## Funding Support and Author Disclosures

The authors have reported that they have no relationships relevant to the contents of this paper to disclose.

**Take-Home Messages**

• AMI cardiogenic shock refractory to conventional therapies requires continuous reassessment as well as an awareness of a differential diagnosis, which includes left ventricular outflow tract obstruction, especially in the presence of left anterior descending artery–related AMI.
• Echocardiography is an important noninvasive modality to promptly diagnose mechanical complications of AMI, including left ventricular outflow tract obstruction, allowing for appropriate management.

## Appendix

Video 1 Left Coronary Artery Angiogram Ninety-percent calcific stenosis in proximal and 95% thrombotic stenosis of the mid-left anterior descending artery. Minor luminal irregularities in left circumflex.

Video 2 Right Coronary Artery Angiogram Minor luminal irregularities in the right coronary artery.

Video 3 Left Coronary Artery Angiogram TIMI 3 flow through proximal and mid-left anterior descending artery after percutaneous coronary intervention with drug-eluting stents. TIMI = thrombolysis in myocardial infarction.

Video 4 Transthoracic Echocardiogram 4-Chamber View With Color Doppler Severe left ventricular systolic dysfunction with severe hypokinesis in the apical and mid-segments and hypercontractility of the basal segments.

Video 5 Transthoracic Echocardiogram Parasternal Long-Axis View With Color Doppler Turbulent jet through the left ventricular outflow tract.

Video 6 Transthoracic Echocardiogram 4-Chamber View With Ultrasound-Enhancing Agent Multiple apical thrombi seen.

Video 7 Transthoracic Echocardiogram Parasternal Long-Axis View at 1-Month Follow-Up No evidence of systolic anterior motion of the mitral valve.

Video 8 Transthoracic Echocardiogram 4-Chamber View at 1-Month Follow-Up Improved left ventricular systolic function and resolution of left ventricular thrombi.
